# Supplementary material for: Blood volume sensitive laminar fMRI with VASO in human hippocampus: Capabilities and biophysical challenges at clinical 7T scanners
Source: bioRxiv. 2025 Aug 29:2025.08.25.672075. Preprint. [Version 1] doi: 10.1101/2025.08.25.672075 (PMC12443046; doi:10.1101/2025.08.25.672075)
Supplement: 1 [file NIHPP2025.08.25.672075V1-supplement-1.pdf]

bioRxiv preprint doi: https://doi.org/10.1101/2025.08.25.672075; this version posted August 29, 2025. The copyright holder for this preprint (which was not certified by peer review) is the author/funder, who has granted bioRxiv a license to display the preprint in perpetuity. It is made available under aCC-BY 4.0 International license.

## Supplementary Materials

| **Participants' ID** | **Sessions** | **Acquired runs** | **Discarded runs due to motion artifacts** | **Total analyzed runs** |
|---|---|---|---|---|
| **001** | 3 | 12 | 3 | 9 |
| **002** | 1 | 3 | 2 | 1 |
| **003** | 1 | 3 | 1 | 2 |
| **004** | 1 | 4 | 1 | 3 |
| **005** | 1 | 3 | 0 | 3 |
| **006** | 2 | 7 | 3 | 4 |

**Supplementary Table 1.** Overview of the number of acquired sessions and functional runs from six participants whose data were analyzed for part 2 of the current study, focused on validating the HC-tailored VASO sequence using an autobiographical memory task.

bioRxiv preprint doi: https://doi.org/10.1101/2025.08.25.672075; this version posted August 29, 2025. The copyright holder for this preprint (which was not certified by peer review) is the author/funder, who has granted bioRxiv a license to display the preprint in perpetuity. It is made available under aCC-BY 4.0 International license.

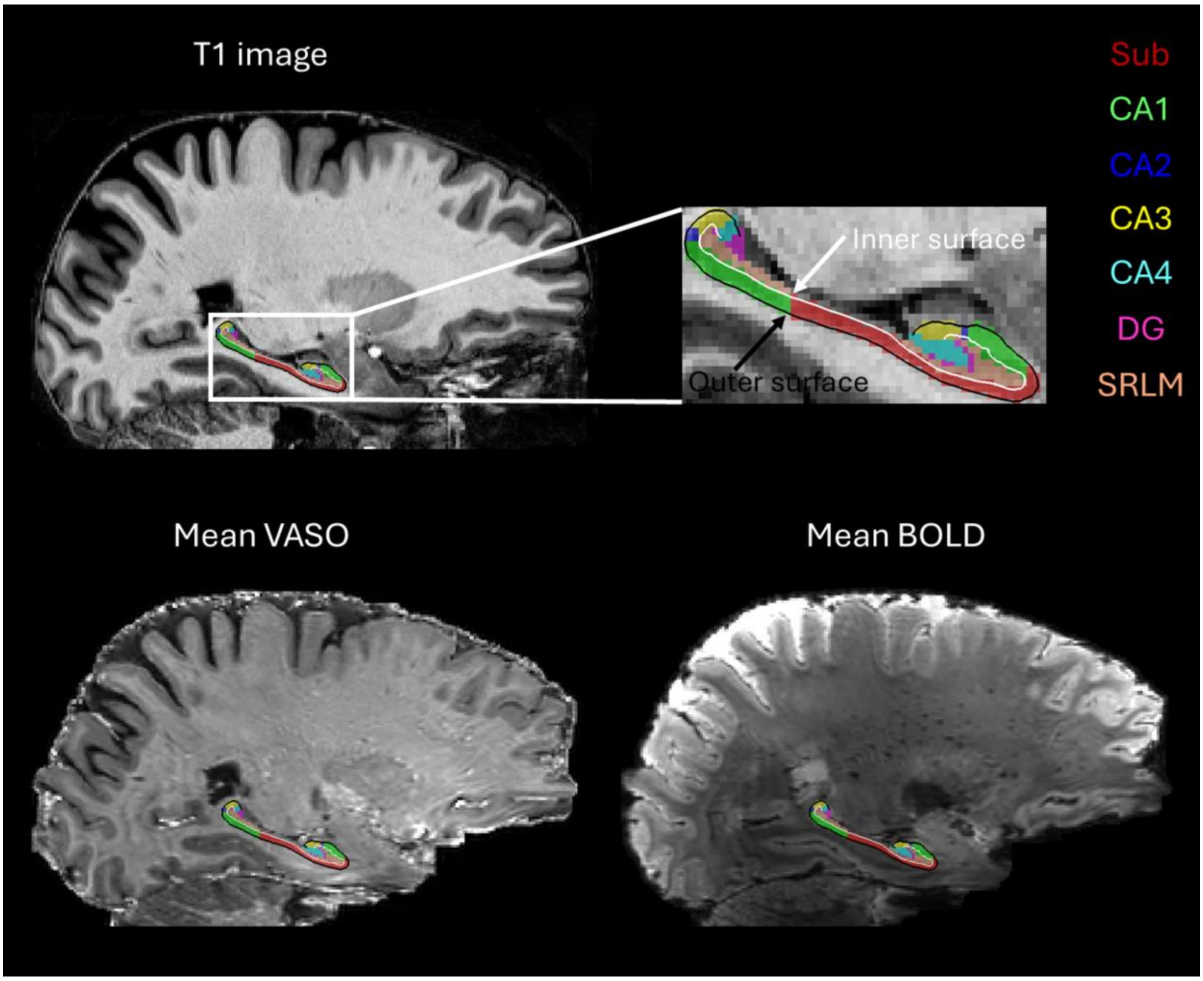


**Supplementary Figure 1.** Hippocampal subfield segmentation and surface boundary delineation overlaid on a T1-weighted image from one participant. The bottom panel is the same as above but displayed on co-registered mean VASO and BOLD images. Subfield labels and inner/outer surfaces were generated using HippUnfold. Sub = subiculum, CA1-CA4 = cornu ammonis areas 1 to 4. DG = dentate gyrus, SRLM = stratum radiatum lacunosom moleculare.

bioRxiv preprint doi: https://doi.org/10.1101/2025.08.25.672075; this version posted August 29, 2025. The copyright holder for this preprint (which was not certified by peer review) is the author/funder, who has granted bioRxiv a license to display the preprint in perpetuity. It is made available under aCC-BY 4.0 International license.

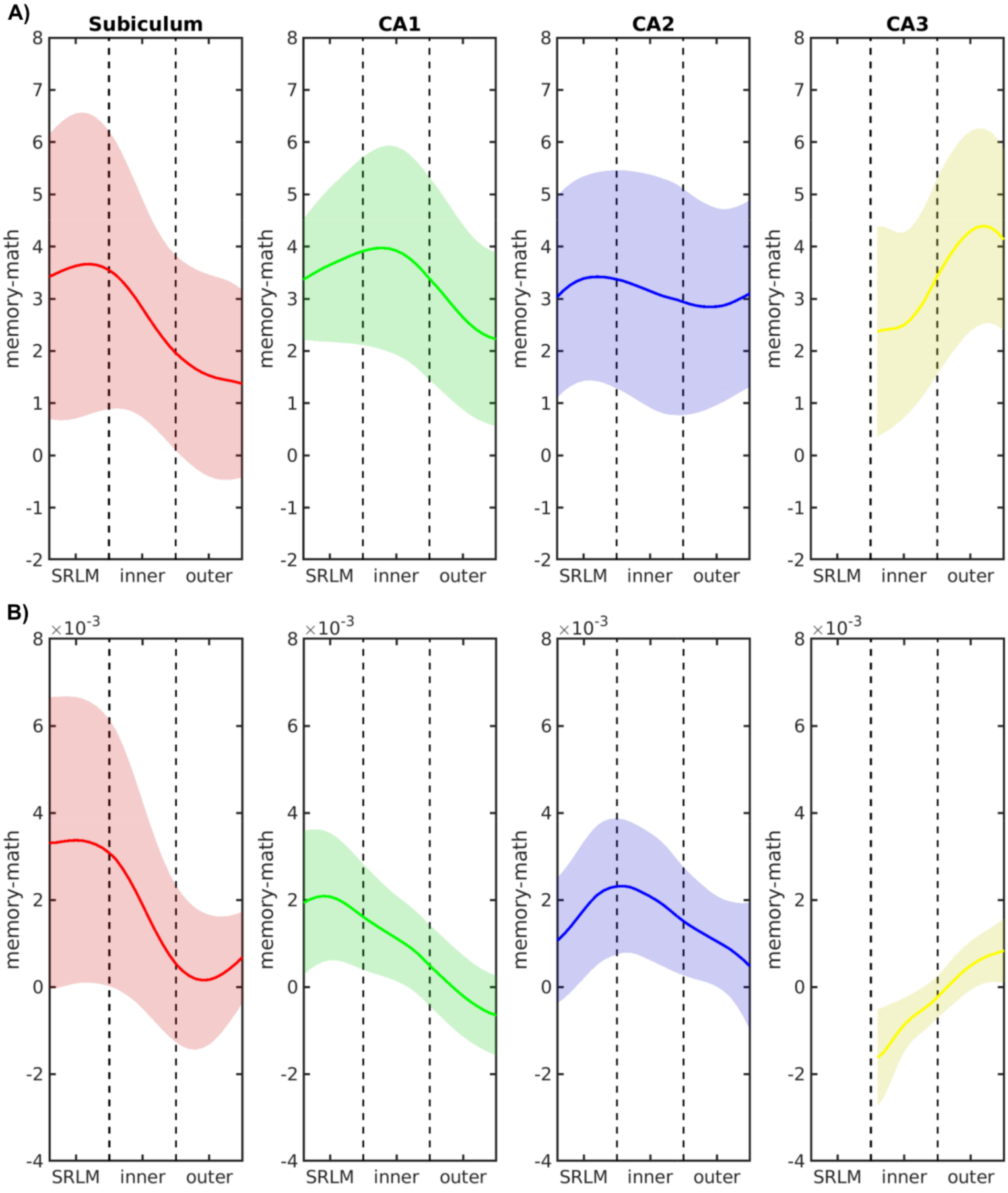


**Supplementary Figure 2.** Laminar profiles of HC subfields for memory vs math contrast (non-transformed). **A)** BOLD signal modulations across the depths of subfields **B)** VASO signal modulations. The laminar profiles of both BOLD and VASO are largely consistent with the patterns observed in z-transformed memory vs. math contrast (see Figure.5).
